# Electrically Evoked Auditory Brainstem Responses in Children Fitted with Hearing Aids Prior to Cochlear Implantation

**DOI:** 10.1177/23312165221148846

**Published:** 2023-01-11

**Authors:** Li Chen, Jun-Ge Zhang, Han-Yu Zhu, Xiao-Yan Hou, Zheng-Quan Tang, Jing-Wu Sun, Jia-Qiang Sun, Xiao-Tao Guo

**Affiliations:** 1 Department of Otolaryngology-Head and Neck Surgery, The First Affiliated Hospital of USTC, Division of Life Sciences and Medicine, University of Science and Technology of China, Hefei, Anhui, 230001, China; 2 Wannan Medical College, Anhui Provincial Hospital, Hefei, Anhui, 230001, China; 3 School of Life Sciences, 12487 Anhui University, Hefei, Anhui, 230601, China; 4 CAS Key Laboratory of Brain Function and Diseases, School of Life Sciences, University of Science and Technology of China, Hefei, Anhui, 230027, China

**Keywords:** cochlear implantation, electrically evoked auditory brainstem response, hearing aid, inner ear malformation

## Abstract

This study investigates the effect of hearing aid use on the peripheral auditory pathways in children with sensorineural hearing loss prior to cochlear implantation, as revealed by the electrically evoked auditory brainstem response (EABR). Forty children with hearing aids were recruited. Half of them had normal inner ear structures and the other half had inner ear malformations (IEMs). The EABR was evoked by electrically stimulating the round window niche (RWN) and round window membrane (RWM) during the cochlear implantation operation. The onset age of hearing aid use was significantly correlated with the peak latencies, but not amplitudes, of the wave III (eIII) and wave V (eV). Higher EABR thresholds were found for RWN stimulation than for RWM stimulation and in the children with IEMs than in those without IEMs. Our study provides neurophysiological evidence that earlier use of hearing aids may ameliorate physiological functions of the peripheral auditory pathway in children with and without IEMs. The EABR evoked by the electrical stimulation at RWM is more sensitive compared with that at RWN for evaluating functions of the auditory conduction pathway.

## Introduction

The development of the auditory system depends to a large extent on sufficient auditory input during the sensitive period, i.e., a period in which the auditory system requires external stimulation to develop skills for auditory processing. Auditory deprivation during the sensitive period will adversely affect many aspects of cortical maturation, hinder the development of auditory pathways, and result in decreased behavioral performance and deficits of spoken language acquisition (Halliday et al., 2017; Ruben & Rapin, 1980; Sharma et al., 2015). In the case of long-term deprivation of auditory input, not only is the bottom-up ability of information processing is reduced, but the integration of bottom-up and top-down information in auditory cortex is also impaired (Kral, 2007). Therefore, it is beneficial to the development of the auditory cortex in children with hearing loss to receive intervention with auditory input at an early stage, especially within the sensitive period. A hearing aid or a cochlear implant (CI) can help people with hearing loss improve auditory sensation. The hearing aid amplifies the sound intensity, but the CI bypasses damaged hair cells in the cochlea and directly stimulates the auditory nerve with electrical pulses (Carlson, 2020; Kral et al., 2019; Lenarz, 2017). Although previous studies have reported that earlier implantation can result in better hearing and speech performance (Holden et al., 2013; McKinney, 2017; Yoshinaga-Itano et al., 2018), many children with hearing loss may receive a CI at a relatively late stage, due to child variables and/or family factors, such as progressive hearing loss and socio-economic status (Dettman et al., 2016; Fitzpatrick et al., 2015). Although wearing a hearing aid before the CI surgery likely provides only a weak input, the auditory sensation from hearing aids in infants with severe or profound hearing loss could still provide limited auditory input that could be beneficial.

The effect of hearing aid use on functions of the auditory pathway has been assessed by using behavioral tests and cortical evoked responses. Relevant evidence demonstrates that hearing aids can help patients with hearing loss improve their speech performance and reduce auditory processing effort (Chen et al., 2010; Giroud et al., 2017). Imaging evidence indicates that hearing aid use can inhibit cross-modal reorganization induced by early auditory deprivation (Shiell et al., 2015). These results suggest that the auditory input by hearing aids benefit the development of the central auditory system. However, direct evidence for relationships between hearing aid use and the development of the peripheral auditory pathway is still lacking. The sensitive periods of peripheral and central auditory systems are different. Relative to the auditory cortex, the development of the brainstem occurs earlier and depends less on auditory experience (Long et al., 2018). Whether early and long-term use of hearing aids benefits the development of the peripheral auditory pathway remains unclear.

The electrically evoked auditory brainstem response (EABR) is an objective and effective method to evaluate functions of the auditory conduction pathway up to the level of the brainstem (Wang et al., 2015; Zhang et al., 2021). It can be recorded from the scalp within the first 10 ms after electrically stimulating the auditory nerve. The EABR can be evoked by extracochlear electrical stimulation, e.g., at the round window niche (RWN) (Causon et al., 2019) or via intracochlear CI stimulation (Firszt et al., 2002). The electrically induced wave III (eIII) and wave V (eV) are easily identified compared with the other EABR components (Adunka et al., 2006). The origins of the EABR components are basically similar to those of the auditory brainstem response (ABR) evoked by acoustic stimuli, and eIII is thought to be generated in the superior olivary nucleus and eV in the inferior colliculus (van den Honert & Stypulkowski, 1986). For normal-hearing children, evidence has shown that the peak latencies of waves I and II (eI and eII) are similar for infants (40 weeks conceptional age) and adults (Ponton et al., 1996). However, eIII remains delayed into the postnatal period compared with the adult response. Waves IV and V also have different maturational time courses (Moore et al., 1996). The III-IV interval reflecting synaptic transmission time gradually shortens until 3 years of age, but the IV-V interval reflecting axonal conduction time is adult-like at term birth. It is noteworthy that these peripheral maturational processes are affected by auditory deprivation. Thai-Van et al. (2002) found a longer EABR latency from the ear with a longer deafness duration, indicating the negative effect of long-term deafness on neural transmission. This finding also highlights the possibility that auditory experience from an early-implanted CI can promote sound exposure-dependent maturational processes.

In this study, we investigated the effect of hearing aid use on physiological functions of the auditory pathway in hearing-impaired children with and without inner ear malformations (IEMs) by using the EABR. The EABR was evoked by electrical stimulation at RWN and round window membrane (RWM) during the operation to implant the CI. We hypothesized that hearing aid use prior to CI activation could promote the development of the auditory brainstem pathway. Therefore, we predicted that children with earlier or longer use of hearing aids would show lower EABR thresholds and/or shorter eIII and eV latencies. Exploratory analyses further compared the EABR thresholds and latencies for two stimulation sites (RWN and RWM) in two groups (children with and without IEMs).

## Materials and Methods

### Participants

Forty children (21 males, mean age  ±  standard deviation (SD): 5.09  ±  3.79 years old) with sensorineural hearing loss who received their first CI in our hospital from September 2018 to June 2020 were included in this study. These children were right-handed according to an assessment with the Edinburgh Handedness Inventory (Oldfield, 1971). They started to use hearing aids at a mean age of 2.30  ±  1.21 years old, and had used hearing aids with a mean duration of 2.79  ±  3.26 years and for at least 4 h per day in their daily life. These children had auditory responses to environmental sounds during the initial period of hearing aid fitting. To confirm the effectiveness of hearing aid fitting in the daily life, their auditory performance was reexamined by the Meaningful Auditory Integration Scale (MAIS) and Categories of Auditory Performance (CAP) at least every 8 months. The MAIS includes 10 questions reflecting children's confidence in hearing devices, auditory sensitivity and ability to connect sounds with meaning. The highest score is 40 and indicates the best performance for meaningful sound use in everyday situations. The CAP is an eight-score hierarchical scale that evaluates receptive auditory abilities and ranges from no awareness of environmental sounds (1 score) to telephone use with a familiar talker (8 scores). When hearing aid outcomes were poor and the ABR thresholds estimated by the click and 500-Hz tone burst were above 90 dB nHL, the hearing-impaired child received a CI. Before the CI surgery, the ABR, 40-Hz auditory evoked potential, multi-frequency steady state potential (MFSSP), distortion product otoacoustic emission (DPOAE) and acoustic impedance had been performed to confirm profound sensorineural hearing loss (hearing threshold ≥90 dB nHL). The 40-Hz auditory evoked potential (Lynn et al., 1984) and MFSSP (Johnson & Brown, 2005) tests were performed for hearing threshold estimation using the 500-Hz tone burst and sinusoidally amplitude modulated tones (1, 2 and 4 kHz), respectively. Only 24 children finished the pure-tone audiometry and their unaided pure tone averages (averaged over 0.25, 0.5, 1, 2, 4 and 8 kHz) were above 90 dB HL. Participants who had a mental disability, intracranial lesions or head trauma were excluded from this study. Of children in our study, 20 had IEMs assessed by computerized tomography (CT) and magnetic resonance imaging (MRI) according to previously published criteria (Sennaroglu & Bajin, 2017). Detailed information for all children is provided in Table 1. All procedures performed in this study involving human participants were in accordance with the ethical standards of the institutional and/or national research committee and with the 1964 Helsinki declaration and its later amendments or comparable ethical standards. The protocols and experimental procedures in the present study were reviewed and approved by the Anhui Provincial Hospital Ethics Committee. Each participant's guardians provided written informed consent.

**Table 1.** Demographic Information of Patients.

Subjects	Sex	Side of test	Age at test (years)	Side of HAs	Onset of HAs (years)	Duration of HAs (years)	Inner ear structure	Etiology	ABR threshold at test (dB nHL)
Patients without inner ear malformations									
1	F	R	4.5	Bi	2.5	2	Normal	Gene	>90
2	F	R	2	Bi	1	1	Normal	Unknown	>95
3	M	R	4.833	Bi	0.833	4	Normal	Unknown	>95
4	M	L	3	Bi	2.5	0.5	Normal	Unknown	>90
5	M	L	14	Bi	3	11	Normal	Unknown	>90
6	M	L	2.5	Bi	1.5	1	Normal	Unknown	>95
7	F	R	5	Bi	1.5	3.5	Normal	Gene	>95
8	F	R	5	Bi	4.417	0.583	Normal	Unknown	>95
9	F	L	8	Bi	4	4	Normal	Unknown	>90
10	M	L	5	Bi	4	1	Normal	Gene	>90
11	M	R	3.833	Bi	2.833	1	Normal	Gene	>95
12	F	L	1	Bi	0.75	0.25	Normal	Gene	>95
13	M	R	2.417	Bi	1.917	0.5	Normal	Unknown	>95
14	F	L	2.167	Bi	2	0.167	Normal	Unknown	>95
15	F	R	14	R	5	9	Normal	Gene	>95
16	F	L	2.833	Bi	1.833	1	Normal	Unknown	>95
17	M	R	5	Bi	4	1	Normal	Unknown	>95
18	F	R	1.583	Bi	1.333	0.25	Normal	Unknown	>95
19	F	R	13.417	Bi	3.417	10	Normal	Unknown	>95
20	M	R	1.83	Bi	1.663	0.167	Normal	Gene	>95
Patients with inner ear malformations									
1	M	L	2	Bi	1.75	0.25	LVAS	LVAS	>95
2	F	L	3.5	Bi	0.5	3	IP-II	IP-II	>95
3	M	R	8	Bi	3	5	LVAS	LVAS	>95
4	M	R	3	Bi	2	1	LVAS	LVAS	>95
5	M	R	5	Bi	3	2	LVAS	LVAS	>95
6	M	R	1.667	Bi	0.667	1	LVAS	LVAS	>95
7	F	R	4	Bi	2	2	CAS	CAS	>95
8	F	R	6.833	Bi	2.833	4	LVAS	LVAS	90
9	F	L	5.333	Bi	4.333	1	LVAS	LVAS	>95
10	M	L	3.75	Bi	1.75	2	LVAS & IP-III	LVAS & IP-III	>90
11	M	L	5.583	Bi	2.583	3	LVAS	LVAS	>95
12	M	L	3	Bi	2	1	CAS & IACS	CAS & IACS	>95
13	M	R	13	Bi	1	12	CAS & IACS	CAS & IACS	>95
14	M	R	1	Bi	0.5	0.5	LVAS & IP-III	LVAS & IP-III	>95
15	F	L	3	Bi	1	2	LVAS	LVAS	95
16	F	L	14	Bi	4	10	LVAS	LVAS	>95
17	M	R	4	Bi	3.5	0.5	LVAS	LVAS	>95
18	F	R	4.75	Bi	1.75	3	LVAS	LVAS	>95
19	F	R	9	Bi	3	6	LVAS	LVAS	>95
20	M	R	1.25	Bi	1	0.25	LVAS	LVAS	>95

ABR = auditory brainstem response; Bi = bilateral; CAS = cochlear aperture stenosis; F = female; HA = hearing aid; HL = hearing level; IACS = inner auditory canal stenosis; IP-II/III = incomplete partition type II/III; L = left; LVAS = large vestibular aqueduct syndrome; M = male; R = right.

### EABR Recording

The EABR was recorded by Neuro-Audio NET1.0.103.3. (Neurosoft, Ivanovo, Russia). The recording electrode, the ground electrode and the reference electrode were body surface button electrodes and were placed in the middle of the forehead, between the eyebrows, and about 1 cm in front of the tragus of the operative ear, respectively. The electrical stimulation was generated from an EMG external electric stimulator (Neurosoft, Ivanovo, Russia). The facial nerve stimulation probe (Medtronic, Minneapolis, USA) was selected as the stimulation positive electrode. Its surface was coated with Parylene insulating coating except for the exposed tail end of the electrode and the diameter was 500 *μ*m. A stainless steel needle electrode was used as the reference electrode and was placed in the coarse protuberance of the occipital bone at the operation side. The EABR output signal was filtered online with a band-pass of 0.1–3 kHz and was averaged from 512 sweeps at each stimulus level with a time window of 15 ms. The electrical pulse was the alternating wave with 100-*μ*s duration and was delivered at a rate of 21 Hz. Electrode impedances were less than 3 kΩ.

All surgical procedures were performed via a mastoidectomy. Posterior tympanotomy was performed through the facial recess. Meticulous hemostasis could be achieved by using diamond burs. After the RWN was exposed, we injected patients with muscle relaxant cis-atracurium at 0.5 mg/kg according to the body weight to reduce the interference of muscle activity from EABR signals. During the first EABR recording, the stimulation probe was placed on the surface of the RWN. Then, a diamond bur was used to remove the RWN and maximally expose the RWM. We performed the second EABR recording by placing the stimulation probe on the surface of the RWM. The initial electrical stimulation intensity for the EABR was 2.0 mA. To assess the EABR threshold, namely the minimum stimulation intensity eliciting eIII or eV, we increased or decreased the stimulation intensity in a first step of 0.5 mA followed by a smaller step of 0.1 mA until the eIII or eV appeared or disappeared. The maximum stimulation intensity was 3.0 mA. The EABR waveform for each stimulation intensity was averaged by 512 epochs and detected visually. Each test of the EABR for the RWN or RWM stimulation lasted 3–5 min.

### Data Processing and Analysis

The eIII and eV were marked by two observers blinded to the information of each child. The EABR thresholds (RWN: *r*  =  0.989; RWM: *r*  =  0.996), eIII latencies (RWN: *r*  =  0.806; RWM: *r*  =  0.861) and eV latencies (RWN: *r*  =  0.804; RWM: *r*  =  0.816) marked by the first observer showed high correlations with the second. Two criteria for identifying the eIII and eV were that the two components should be reproducible at least at two different stimulation intensities and the difference in peak latencies of each component should be ≤0.3 ms between adjacent stimulation intensities. One child without IEMs and one with IEMs showed no robust eIII and eV, and these two children were excluded from further analysis. SPSS Statistics V.24 (IBM, Somers, NY) was used for statistical analysis. The eIII and eV latencies for each individual were determined at a stimulation intensity of 2.0 mA when the EABR threshold was ≤2.0 mA or at the stimulation intensity of the EABR threshold when the threshold was >2.0 mA. Differences in the EABR thresholds and latencies between the two stimulation methods and the two groups were analyzed by using a two-way analysis of variance (ANOVA) with stimulation method (RWN and RWM) as the within-subjects factor and group (children with and without IEMs) as the between-subjects factor. The Greenhouse–Geisser adjustment was applied when the variance sphericity assumption was not satisfied. A Pearson correlation test was used to assess the correlations between the onset age and duration of hearing aid use and the EABR threshold, eIII latency, and eV latency for all participants. The *p* values were corrected for multiple comparisons using the false discovery rate (FDR). All values in this study are expressed as mean  ±  SD.

## Results

### EABR Thresholds and Latencies for RWN and RWM Stimulation in Children without and with IEMs

**Figure 1.**
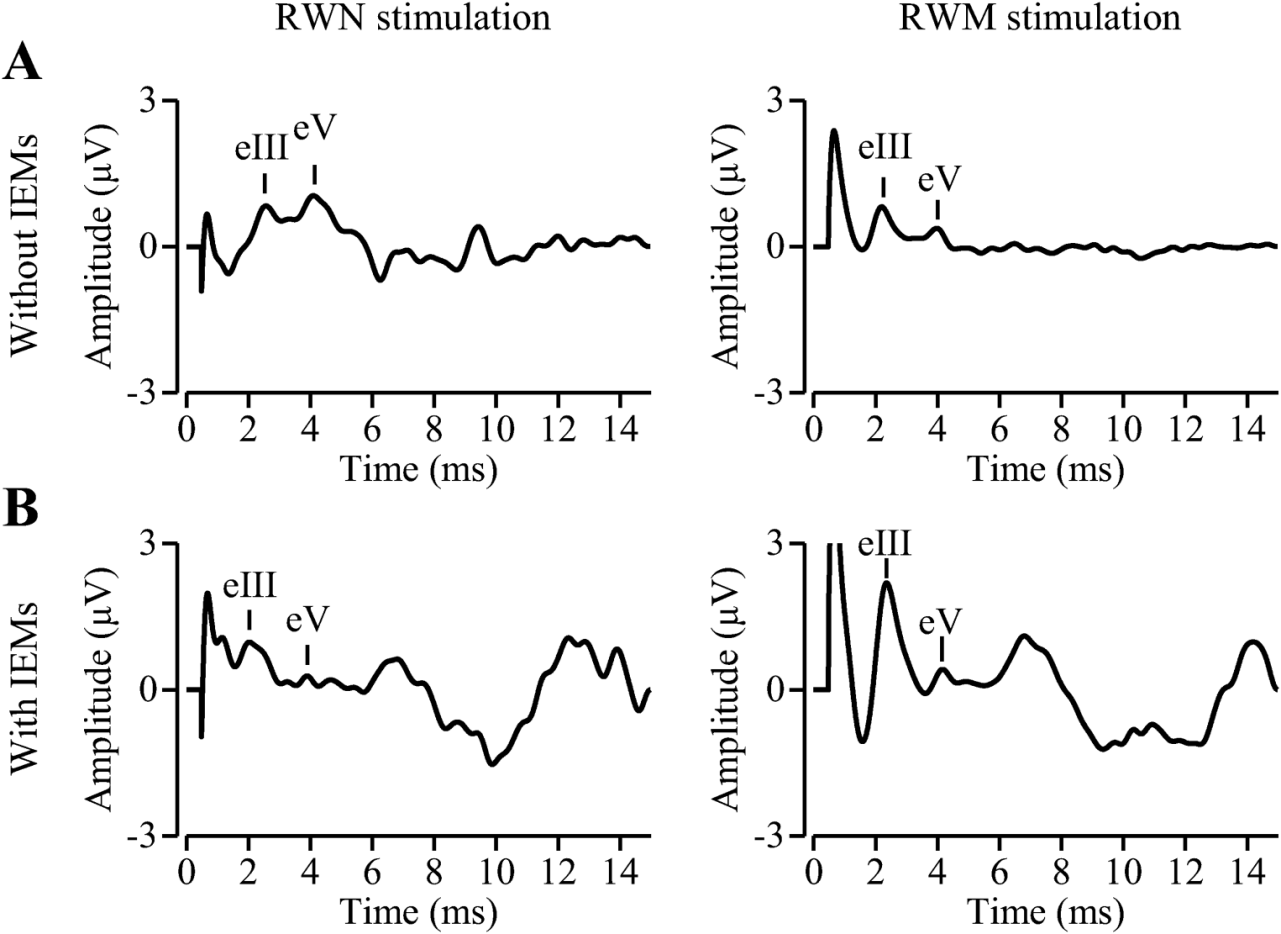
Sample waveforms of electrically evoked auditory brainstem responses (EABRs) evoked by electrical stimulation at the round window niche (RWN) and round window membrane (RWM) from (A) Subject #2 without inner ear malformations (IEMs) and (B) Subject #3 with IEMs. eIII, wave III. eV, wave V.

**Figure 2.**
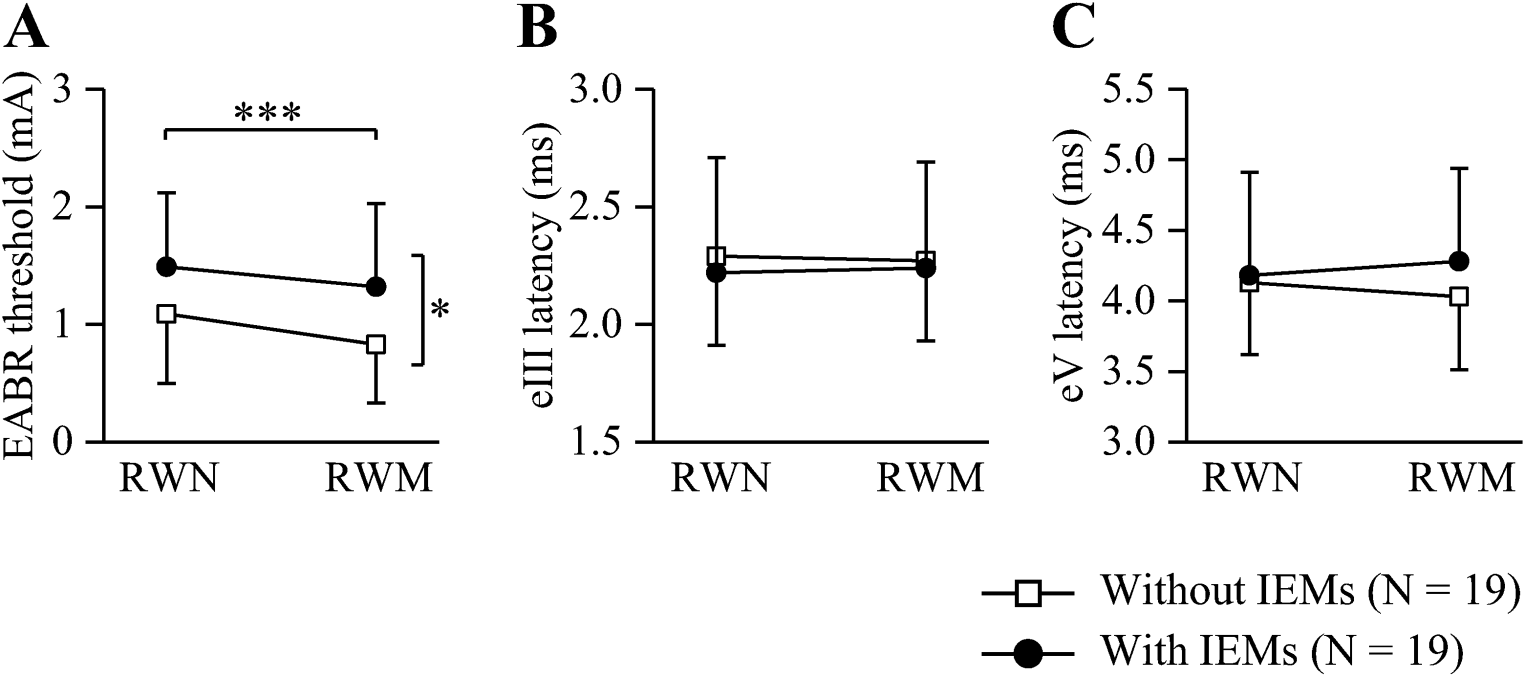
The thresholds, wave III (eIII) latencies, and wave V (eV) latencies of electrically EABRs evoked by electrical stimulation at the RWN and RWM in children with and without inner ear malformations (IEMs). (A) The EABR thresholds were significantly higher for RWN stimulation than for RWM stimulation and in children with IEMs than in those without IEMs. There was no significant difference in (B) eIII or (C) eV peak latencies between RWN and RWM stimulation or between these two groups. ***p < 0.001, *p < 0.05. Vertical bars represent the standard deviation.

Sample waveforms of EABRs at a stimulation intensity of 2.0 mA from two children are shown in Figure 1. The EABR thresholds in children without and with IEMs were 1.09  ±  0.59 mA and 1.49  ±  0.63 mA for RWN stimulation, and 0.83  ±  0.50 mA and 1.32  ±  0.71 mA for RWM stimulation, respectively. A two-way ANOVA showed main effects of stimulation [*F*(1,36)  =  13.380, *p* < 0.001] and group [*F*(1,36)  =  5.510, *p*  =  0.025]. There was no significant interaction between these two factors [*F*(1,36)  =  0.437, *p*  =  0.513]. The EABR thresholds were significantly higher for RWN stimulation than for RWM stimulation and in children with IEMs than in those without IEMs (Figure 2A).

The eIII peak latencies in children without and with IEMs were 2.29  ±  0.38 ms and 2.22  ±  0.49 ms for RWN stimulation, and 2.27  ±  0.34 ms and 2.24  ±  0.45 ms for RWM stimulation, respectively. The eV peak latencies in children without and with IEMs were 4.13  ±  0.51 ms and 4.18  ±  0.73 ms for RWN stimulation, and 4.03  ±  0.52 ms and 4.28  ±  0.66 ms for RWM stimulation, respectively. For eIII and eV peak latencies, there was no main effect of stimulation [eIII: *F*(1,36) < 0.001, *p*  =  0.996; eV: *F*(1,36) < 0.001, *p*  =  0.995], group [eIII: *F*(1,36)  =  0.168, *p*  =  0.684; eV: *F*(1,36)  =  0.687, *p*  =  0.413] or significant interaction between these two factors [eIII: *F*(1,36)  =  0.125, *p*  =  0.726; eV: *F*(1,36)  =  1.430, *p*  =  0.240] (Figure 2B and C).

### Correlations between EABR Threshold and Latency and Onset Age and Duration of Hearing Aid Use

**Figure 3.**
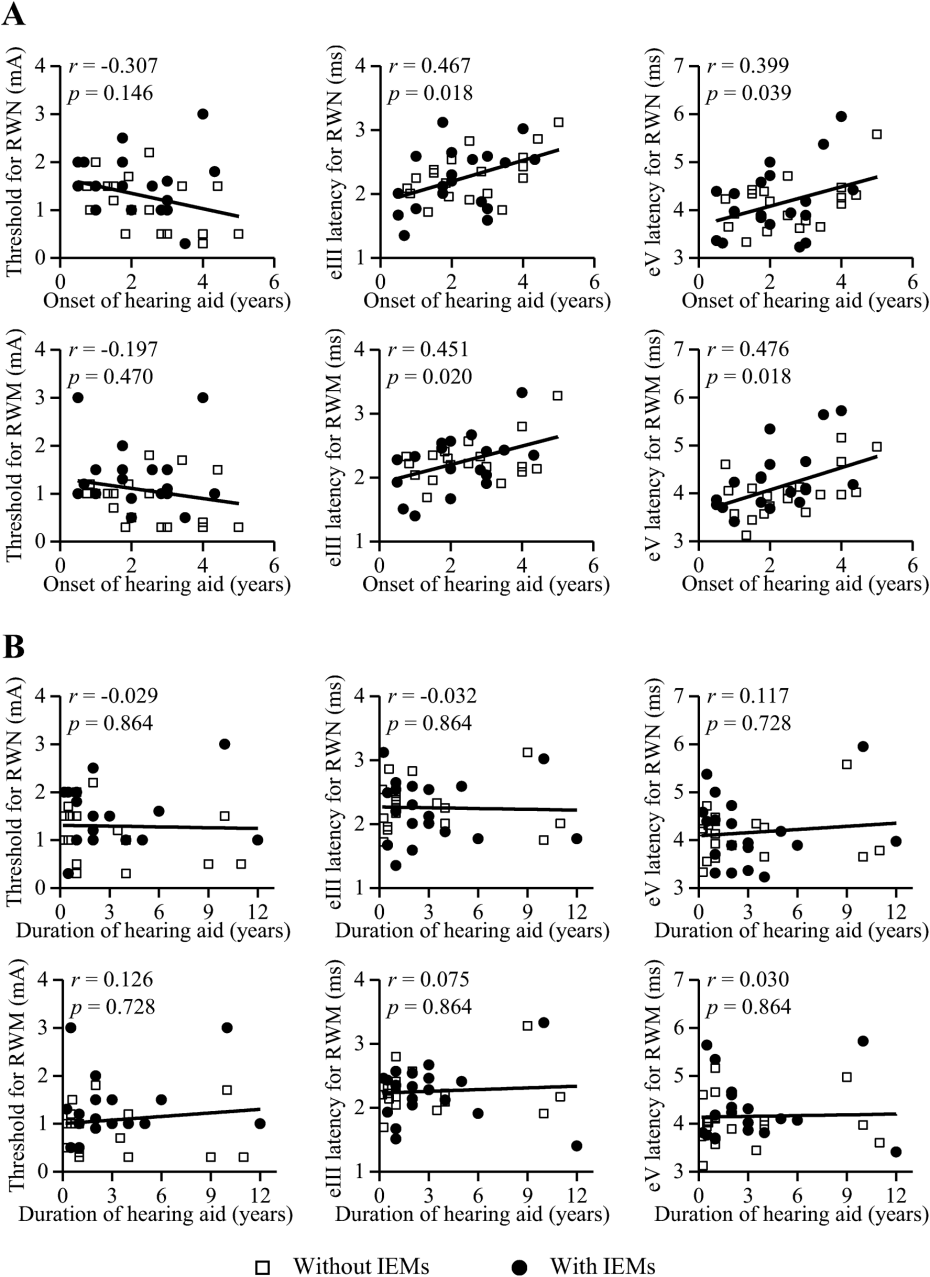
Correlations between the threshold and latency of the electrically EABR and onset age and duration of hearing aid use for all participants. (A) The onset age of hearing aid use was significantly positively correlated with the peak latencies of the wave III (eIII) and wave V (eV) but not with the thresholds. (B) No significant correlation between the duration of hearing aid use and the threshold, eIII latency or eV latency was found. IEM, inner ear malformation. RWM, round window membrane. RWN, round window niche.

Correlations were examined between the onset age and duration of hearing aid use and the EABR threshold, eIII latency, and eV latency for all participants. The onset age of hearing aid use was significantly positively correlated with the eIII (RWN: *r*  =  0.467, *p*  =  0.018; RWM: *r*  =  0.451, *p*  =  0.020) and eV latencies (RWN: *r*  =  0.399, *p*  =  0.039; RWM: *r*  =  0.476, *p*  =  0.018) but not with the EABR thresholds (RWN: *r*  =  −0.307, *p*  =  0.146; RWM: *r*  =  -0.197, *p*  =  0.470) (Figure 3A). No significant correlations were found between the duration of hearing aid use and the EABR threshold (RWN: *r*  =  -0.029, *p*  =  0.864; RWM: *r*  =  0.126, *p*  =  0.728), eIII latency (RWN: *r*  =  −0.032, *p*  =  0.864; RWM: *r*  =  0.075, *p*  =  0.864), or eV latency (RWN: *r*  =  0.117, *p*  =  0.728; RWM: *r*  =  0.030, *p*  =  0.864) (Figure 3B).

## Discussion

In this study, we examined the effect of hearing aid use on the peripheral auditory pathway in children with sensorineural hearing loss by recording the intraoperative EABRs. We found positive correlations between the onset age of hearing aid use and the peak latencies of the eIII and eV. Furthermore, higher EABR thresholds were found for RWN stimulation than for RWM stimulation and in children with IEMs than in those without IEMs. Our neurophysiological results suggest that earlier use of hearing aids can ameliorate physiological functions of the peripheral auditory pathway in children with sensorineural hearing loss with and without IEMs. Furthermore, the EABR evoked by the electrical stimulation at RWM is more sensitive compared with that at RWN for evaluating functions of the auditory conduction pathway.

The EABR eIII and eV are less affected by electrical artifacts compared with eI and eII and are therefore usually used as neurophysiological indicators in implanted individuals. The two later components (eIII and eV) indicate that the signal has reached the brainstem at a higher level along the auditory pathway (the superior olivary nucleus and the inferior colliculus). We previously reported that the EABR response rates were 80% and 55% for patients with no IEMs and those with Mondini malformation, respectively (Zhu et al., 2022). The high EABR response rate (95%) found in the present study suggests that a coarse neural pathway still develops in children with severe-to-profound hearing loss (Lassaletta et al., 2017). We further found significantly positive correlations between the onset age of hearing aid use and the eIII and eV peak latencies. Shorter EABR latency reflects decreased neural conduction times (Gordon et al., 2002, 2003; Thai-Van et al., 2007). Therefore, the results demonstrate that earlier auditory input via hearing aid fitting can ameliorate the auditory conduction functions in deaf children. Underlying mechanisms may include myelination and/or an improvement in synaptic efficacy. Our findings are consistent with previous ones which suggest that the EABR is sensitive to the duration of deafness (Guiraud et al., 2007; Thai-Van et al., 2002). Interesting, no correlation was observed between the duration of hearing aid use and the EABR latency, which could be explained by the progressive nature of the hearing loss that was part of the inclusion criteria for the study. It should be noted that only participants who had residual hearing and auditory responses to environmental sounds with the help of hearing aids were recruited because this study investigates the effect of hearing aid use on functions of the peripheral auditory pathway. These participants finally had profound hearing loss (hearing threshold ≥90 dB nHL) and had to select cochlear implantation. Therefore, auditory sensation provided by hearing aids may last for a short period and its positive effect on the development of the auditory pathway is still limited. Furthermore, the progression of hearing loss (e.g., rate of hearing decline) can vary between individual children. These factors may contribute to the finding of no correlation between the duration of hearing aid use and the EABR latency. Although we only found that early use of hearing aids can benefit the development of the auditory brainstem pathway, the effect of long-term use of hearing aids on the auditory conduction functions still cannot be ruled out because we only examined it in children with progressive hearing loss. Relationships between the duration of hearing aid use and the auditory conduction functions should be investigated in patients with stable hearing levels.

Previous electrophysiological studies have shown the positive effect of an early CI on the development of the central auditory system (Dorman et al., 2007; Sharma et al., 2002). Long-term auditory deprivation may cause cross-modal plasticity in prelingually deafened patients, resulting in no improvement in hearing even after implantation and concentrated rehabilitation (Buckley & Tobey, 2011; Lee et al., 2001). Shiell et al. (2015) found that early auditory deprivation alters the connectivity between the auditory and visual cortical areas. However, this cross-modal reorganization can be inhibited by hearing aid use. Here, our findings further suggest that early use of hearing aids can promote the development of the peripheral auditory pathway (as revealed by the EABR) in addition to the auditory cortex. Sounds may stimulate the auditory system via hearing aids and maintain the neural sensitivity to the auditory stimulation, providing the basis for the auditory and speech rehabilitation after implantation.

It is suggested that a hearing aid is fitted as early as possible for the development of the auditory system, which is also supported by our finding that the onset age of hearing aid use was positively correlated with latencies of eIII and eV. However, in this study, children with hearing loss received hearing aids relatively late (at a mean age of 2.3 years old). A late intervention with a hearing aid has also been found in other studies (Rohlfs et al., 2017; Shiell et al., 2015). This can be explained by progressive hearing loss. In this study, the participants with residual hearing had responses to environmental sounds at an early stage, which might result in late identification of hearing loss. These participants finally had very high ABR thresholds (≥90 dB nHL) and selected cochlear implantation.

The EABR can be evoked via intracochlear CI stimulation (Firszt et al., 2002) or by extracochlear electrical stimulation (Causon et al., 2019). Before the CI surgery, the EABR test is usually performed by electrical stimulation at the RWN or promontory and is very significant for estimation of functional integrity of the auditory nerve, especially for patients with IEMs (Gibson & Sanli, 2007; Kileny et al., 2010). However, the current from the stimulating electrode cannot easily spread into the cochlea because of the bone of the RWN. In this study, we recorded the EABRs by electrically stimulating the RWN and RWM. The EABR threshold was significantly lower for RWM stimulation than for RWN stimulation, suggesting that the EABR for electrical stimulation at the RWM is more sensitive and effective for assessing the auditory conduction functions. Moreover, we also found a lower EABR threshold for hearing-impaired children with normal cochlear structures than those with IEMs. It may result from the reduction in the number of and abnormal distribution of the spiral ganglion cells, or poor synchronization of auditory nerve fibers in patients with IEMs. It should be noted that the prognostic value of the intraoperative EABR exists but is limited. The absence of the EABR does not necessarily indicate poor CI outcomes (Nikolopoulos et al., 2000) because the EABR mainly reflects functions of the auditory conduction pathway up to the level of the brainstem and the CI outcomes are also affected by postoperative training.

In conclusion, our EABR findings demonstrate that earlier use of hearing aids can ameliorate physiological functions of the peripheral auditory pathway in pediatric CI candidates. Early hearing aid fitting can promote the development of the auditory system during the sensitive periods, providing the neural basis for speech rehabilitation after a CI is received. Our neurophysiological evidence also suggests that the EABR evoked by the electrical stimulation at RWM may be more sensitive and effective for evaluating auditory conduction functions than stimulation at RWN.

## References

[bibr1-23312165221148846] Adunka O. F. Roush P. A. Teagle H. F. Brown C. J. Zdanski C. J. Jewells V. Buchman C. A. (2006). Internal auditory canal morphology in children with cochlear nerve deficiency. Otology & Neurotology, 27(6), 793–801. 10.1097/01.mao.0000227895.34915.94 16936566

[bibr2-23312165221148846] Buckley K. A. Tobey E. A. (2011). Cross-modal plasticity and speech perception in pre- and postlingually deaf cochlear implant users. Ear and Hearing, 32(1), 2–15. 10.1097/AUD.0b013e3181e8534c 20829699

[bibr3-23312165221148846] Carlson M. L. (2020). Cochlear implantation in adults. New England Journal of Medicine, 382(16), 1531–1542. 10.1056/NEJMra1904407 32294347

[bibr4-23312165221148846] Causon A. O'Driscoll M. Stapleton E. Lloyd S. Freeman S. Munro K. J. (2019). Extracochlear stimulation of electrically evoked Auditory Brainstem Responses (eABRs) remains the preferred Pre-implant auditory nerve function test in an assessor-blinded comparison. Otology & Neurotology, 40(1), 47–55. 10.1097/MAO.0000000000002055 30489452

[bibr5-23312165221148846] Chen X. Liu S. Liu B. Mo L. Kong Y. Liu H. Zhang L. (2010). The effects of age at cochlear implantation and hearing aid trial on auditory performance of Chinese infants. Acta Oto-Laryngologica, 130(2), 263–270. 10.3109/00016480903150528 19680991

[bibr6-23312165221148846] Dettman S. Choo D. Dowell R. (2016). Barriers to early cochlear implantation. International Journal of Audiology, 55(Suppl 2), S64–S76. 10.1080/14992027.2016.1174890 27139125

[bibr7-23312165221148846] Dorman M. F. Sharma A. Gilley P. Martin K. Roland P. (2007). Central auditory development: Evidence from CAEP measurements in children fit with cochlear implants. Journal of Communication Disorders, 40(4), 284–294. 10.1016/j.jcomdis.2007.03.007 17433357 PMC2755241

[bibr8-23312165221148846] Firszt J. B. Chambers R. D. Kraus N. Reeder R. M. (2002). Neurophysiology of cochlear implant users I: Effects of stimulus current level and electrode site on the electrical ABR, MLR, and N1-P2 response. Ear and Hearing, 23(6), 502–515. 10.1097/00003446-200212000-00002 12476088

[bibr9-23312165221148846] Fitzpatrick E. M. Ham J. Whittingham J. (2015). Pediatric cochlear implantation: Why do children receive implants late? Ear and Hearing, 36(6), 688–694. 10.1097/AUD.0000000000000184 26035143 PMC4617290

[bibr10-23312165221148846] Gibson W. P. Sanli H. (2007). Auditory neuropathy: An update. Ear and Hearing, 28(2 Suppl), 102S–106S. 10.1097/AUD.0b013e3180315392 17496659

[bibr11-23312165221148846] Giroud N. Lemke U. Reich P. Matthes K. L. Meyer M. (2017). The impact of hearing aids and age-related hearing loss on auditory plasticity across three months - an electrical neuroimaging study. Hearing Research, 353, 162–175. 10.1016/j.heares.2017.06.012 28705608

[bibr12-23312165221148846] Gordon K. A. Papsin B. C. Harrison R. V. (2002). Auditory brain stem and midbrain development after cochlear implantation in children. Annals of Otology, Rhinology, and Laryngology. Supplement, 189, 32–37. 10.1177/00034894021110s507 12018345

[bibr13-23312165221148846] Gordon K. A. Papsin B. C. Harrison R. V. (2003). Activity-dependent developmental plasticity of the auditory brain stem in children who use cochlear implants. Ear and Hearing, 24(6), 485–500. 10.1097/01.AUD.0000100203.65990.D4 14663348

[bibr14-23312165221148846] Guiraud J. Gallego S. Arnold L. Boyle P. Truy E. Collet L. (2007). Effects of auditory pathway anatomy and deafness characteristics? (1): On electrically evoked auditory brainstem responses. Hearing Research, 223(1-2), 48–60. 10.1016/j.heares.2006.09.014 17157463

[bibr15-23312165221148846] Halliday L. F. Tuomainen O. Rosen S. (2017). Auditory processing deficits are sometimes necessary and sometimes sufficient for language difficulties in children: Evidence from mild to moderate sensorineural hearing loss. Cognition, 166, 139–151. 10.1016/j.cognition.2017.04.014 28577444

[bibr16-23312165221148846] Holden L. K. Finley C. C. Firszt J. B. Holden T. A. Brenner C., Potts L. G., … Skinner M. W. (2013). Factors affecting open-set word recognition in adults with cochlear implants. Ear and Hearing, 34(3), 342–360. 10.1097/AUD.0b013e3182741aa7 23348845 PMC3636188

[bibr17-23312165221148846] Johnson T. A. Brown C. J. (2005). Threshold prediction using the auditory steady-state response and the tone burst auditory brain stem response: A within-subject comparison. Ear and Hearing, 26(6), 559–576. 10.1097/01.aud.0000188105.75872.a3 16377993

[bibr18-23312165221148846] Kileny P. R. Kim A. H. Wiet R. M. Telian S. A. Arts H. A. El-Kashlan H. Zwolan T. A. (2010). The predictive value of transtympanic promontory EABR in congenital temporal bone malformations. Cochlear Implants International, 11(Suppl 1), 181–186. 10.1179/146701010X12671177818669 21756608

[bibr19-23312165221148846] Kral A. (2007). Unimodal and cross-modal plasticity in the ‘deaf’ auditory cortex. International Journal of Audiology, 46(9), 479–493. 10.1080/14992020701383027 17828664

[bibr20-23312165221148846] Kral A. Dorman M. F. Wilson B. S. (2019). Neuronal development of hearing and language: Cochlear implants and critical periods. Annual Review of Neuroscience, 42, 47–65. 10.1146/annurev-neuro-080317-061513 30699049

[bibr21-23312165221148846] Lassaletta L. Polak M. Huesers J. Diaz-Gomez M. Calvino M. Varela-Nieto I. Gavilan J. (2017). Usefulness of electrical auditory brainstem responses to assess the functionality of the cochlear nerve using an intracochlear test electrode. Otology & Neurotology, 38(10), e413–e420. 10.1097/MAO.0000000000001584 29076926

[bibr22-23312165221148846] Lee D. S. Lee J. S. Oh S. H. Kim S. K. Kim J. W., Chung J. K., … Kim C. S. (2001). Cross-modal plasticity and cochlear implants. Nature, 409(6817), 149–150. 10.1038/35051653 11196628

[bibr23-23312165221148846] Lenarz T. (2017). Cochlear implant - state of the art. GMS Current Topics in Otorhinolaryngology, Head and Neck Surgery, 16, Doc04. . 10.3205/cto000143 29503669 PMC5818683

[bibr24-23312165221148846] Long P. Wan G. Roberts M. T. Corfas G. (2018). Myelin development, plasticity, and pathology in the auditory system. Developmental Neurobiology, 78(2), 80–92. 10.1002/dneu.22538 28925106 PMC5773349

[bibr25-23312165221148846] Lynn J. M. Lesner S. A. Sandridge S. A. Daddario C. C. (1984). Threshold prediction from the auditory 40-Hz evoked potential. Ear and Hearing, 5(6), 366–370. 10.1097/00003446-198411000-00009 6510584

[bibr26-23312165221148846] McKinney S. (2017). Cochlear implantation in children under 12 months of age. Current Opinion in Otolaryngology & Head and Neck Surgery, 25(5), 400–404. 10.1097/MOO.0000000000000400 28719394

[bibr27-23312165221148846] Moore J. K. Ponton C. W. Eggermont J. J. Wu B. J. Huang J. Q. (1996). Perinatal maturation of the auditory brain stem response: Changes in path length and conduction velocity. Ear and Hearing, 17(5), 411–418. 10.1097/00003446-199610000-00007 8909889

[bibr28-23312165221148846] Nikolopoulos T. P. Mason S. M. Gibbin K. P. O'Donoghue G. M. (2000). The prognostic value of promontory electric auditory brain stem response in pediatric cochlear implantation. Ear and Hearing, 21(3), 236–241. 10.1097/00003446-200006000-00007 10890732

[bibr29-23312165221148846] Oldfield R. C. (1971). The assessment and analysis of handedness: The Edinburgh inventory. Neuropsychologia, 9(1), 97–113. 10.1016/0028-3932(71)90067-4 5146491

[bibr30-23312165221148846] Ponton C. W. Moore J. K. Eggermont J. J. (1996). Auditory brain stem response generation by parallel pathways: Differential maturation of axonal conduction time and synaptic transmission. Ear and Hearing, 17(5), 402–410. 10.1097/00003446-199610000-00006 8909888

[bibr31-23312165221148846] Rohlfs A. K. Friedhoff J. Bohnert A. Breitfuss A. Hess M. Muller F., … Wiesner T. (2017). Unilateral hearing loss in children: A retrospective study and a review of the current literature. European Journal of Pediatrics, 176(4), 475–486. 10.1007/s00431-016-2827-2 28132094

[bibr32-23312165221148846] Ruben R. J. Rapin I. (1980). Plasticity of the developing auditory system. Annals of Otology, Rhinology and Laryngology, 89(4 Pt 1), 303–311. 10.1177/000348948008900403 7416679

[bibr33-23312165221148846] Sennaroglu L. Bajin M. D. (2017). Classification and current management of inner ear malformations. Balkan Medical Journal, 34(5), 397–411. 10.4274/balkanmedj.2017.0367 28840850 PMC5635626

[bibr34-23312165221148846] Sharma A. Campbell J. Cardon G. (2015). Developmental and cross-modal plasticity in deafness: Evidence from the P1 and N1 event related potentials in cochlear implanted children. International Journal of Psychophysiology, 95(2), 135–144. 10.1016/j.ijpsycho.2014.04.007 24780192 PMC4209331

[bibr35-23312165221148846] Sharma A. Dorman M. F. Spahr A. J. (2002). A sensitive period for the development of the central auditory system in children with cochlear implants: Implications for age of implantation. Ear and Hearing, 23(6), 532–539. 10.1097/00003446-200212000-00004 12476090

[bibr36-23312165221148846] Shiell M. M. Champoux F. Zatorre R. J. (2015). Reorganization of auditory cortex in early-deaf people: Functional connectivity and relationship to hearing aid use. Journal of Cognitive Neuroscience, 27(1), 150–163. 10.1162/jocn_a_00683 25000527

[bibr37-23312165221148846] Thai-Van H. Cozma S. Boutitie F. Disant F. Truy E. Collet L. (2007). The pattern of auditory brainstem response wave V maturation in cochlear-implanted children. Clinical Neurophysiology, 118(3), 676–689. 10.1016/j.clinph.2006.11.010 17223382

[bibr38-23312165221148846] Thai-Van H. Gallego S. Truy E. Veuillet E. Collet L. (2002). Electrophysiological findings in two bilateral cochlear implant cases: Does the duration of deafness affect electrically evoked auditory brain stem responses? Annals of Otology, Rhinology and Laryngology, 111(11), 1008–1014. 10.1177/000348940211101111 12450176

[bibr39-23312165221148846] van den Honert C. Stypulkowski P. H. (1986). Characterization of the electrically evoked auditory brainstem response (ABR) in cats and humans. Hearing Research, 21(2), 109–126. 10.1016/0378-5955(86)90033-x 3754550

[bibr40-23312165221148846] Wang Y. Pan T. Deshpande S. B. Ma F. (2015). The relationship between EABR and auditory performance and speech intelligibility outcomes in pediatric cochlear implant recipients. American Journal of Audiology, 24(2), 226–234. 10.1044/2015_AJA-14-0023 25677645

[bibr41-23312165221148846] Yoshinaga-Itano C. Sedey A. L. Wiggin M. Mason C. A. (2018). Language outcomes improved through early hearing detection and earlier cochlear implantation. Otology & Neurotology, 39(10), 1256–1263. 10.1097/MAO.0000000000001976 30444842

[bibr42-23312165221148846] Zhang J. G. Chen L. Li P. Sun J. W. Guo X. T. Sun J. Q. (2021). Effect of unilateral cochlear implant use on contralateral electrically evoked auditory brainstem responses to round window membrane electrical stimulation. Acta Oto-Laryngologica, 141(6), 588–593. 10.1080/00016489.2021.1906443 33823755

[bibr43-23312165221148846] Zhu H. Y. Chen L. Hou X. Y. Tang Z. Q. Sun J. Q. Sun J. W. Guo X. T. (2022). Electrically evoked auditory brainstem responses in deaf patients with Mondini malformation during cochlear implantation. European Archives of Oto-Rhino-Laryngology, 279(10), 4847–4852. 10.1007/s00405-022-07307-9 35247096

